# Assessment of the feasibility and coverage of a modified universal hearing screening protocol for use with newborn babies of migrant workers in Beijing

**DOI:** 10.1186/1471-2431-13-116

**Published:** 2013-08-08

**Authors:** Beier Qi, Xiaohua Cheng, Hui En, Bo Liu, Shichun Peng, Yong Zhen, Zhenghua Cai, Lihui Huang, Luo Zhang, Demin Han

**Affiliations:** 1 Department of Otolaryngology Head and Neck Surgery, Beijing Tongren Hospital, Capital Medical University, 17 HouGouHuTong, DongCheng District, Beijing, 100005, China; 2 Key Laboratory of Otolaryngology Head and Neck Surgery (Ministry of Education), Beijing Institute of Otolaryngology, 17 HouGouHuTong, DongCheng District, Beijing, 100005, China

**Keywords:** Auditory brainstem response (ABR), Migrant people, Newborn babies, Otoacoustic emissions (OAE), Universal newborn hearing screening (UNHS)

## Abstract

**Background:**

Although migrant workers account for the majority of newborns in Beijing, their children are less likely to undergo appropriate universal newborn hearing screening/rescreening (UNHS) than newborns of local non-migrant residents. We hypothesised that this was at least in part due to the inadequacy of the UNHS protocol currently employed for newborn babies, and therefore aimed to modify the protocol to specifically reflect the needs of the migrant population.

**Methods:**

A total of 10,983 healthy babies born to migrant mothers between January 2007 and December 2009 at a Beijing public hospital were investigated for hearing abnormalities according to a modified UNHS protocol. This incorporated two additional/optional otoacoustic emissions (OAE) tests at 24–48 hours and 2 months after birth. Infants not passing a screening test were referred to the next test, until any hearing loss was confirmed by the auditory brainstem response (ABR) test.

**Results:**

A total of 98.91% (10983/11104) of all newborn children underwent the initial OAE test, of which 27.22% (2990/10983) failed the test. 1712 of the failed babies underwent the second inpatient OAE test, with739 failing again; thus significantly decreasing the overall positive rate for abnormal hearing from 27.22% to 18.36% ([2990–973 /10983)]; p = 0). Overall, 1147(56.87%) babies underwent the outpatient OAE test again after1-month, of whom 228 failed and were referred for the second outpatient OAE test (i.e. 2.08% (228/10983) referral rate at 1month of age). 141 of these infants underwent the referral test, of whom 103 (73.05%) tested positive again and were referred for a final ABR test for hearing loss (i.e. final referral rate of 1.73% ([228-38/10983] at 2 months of age). Only 54 infants attended the ABR test and 35 (0.32% of the original cohort tested) were diagnosed with abnormal hearing.

**Conclusions:**

Our study shows that it is feasible and practical to achieve high coverage rates for screening hearing loss and decrease the referral rates in newborn babies of migrant workers, using a modification of the currently employed UNHS protocol.

## Background

Hearing loss is a major sensory deficiency, which affects audiological development and impairs the quality of life of those affected [1,2]. One in every 1000 newborn babies has a congenital bilateral hearing impairment requiring rehabilitation [3]. As adequate auditory stimulation in early childhood is fundamental for optimal speech and language development as well as for the acquisition of literacy skills, early hearing loss detection and intervention in deaf children are essential [4,5]. This is particularly so within the first three years of birth to prevent severe and irreversible developmental abnormalities of the central auditory system, impairment in language acquisition and speech development in early life, poor educational performance in childhood and adolescence, and poor occupational performance in adulthood [3,6-9].

**Figure 1:**
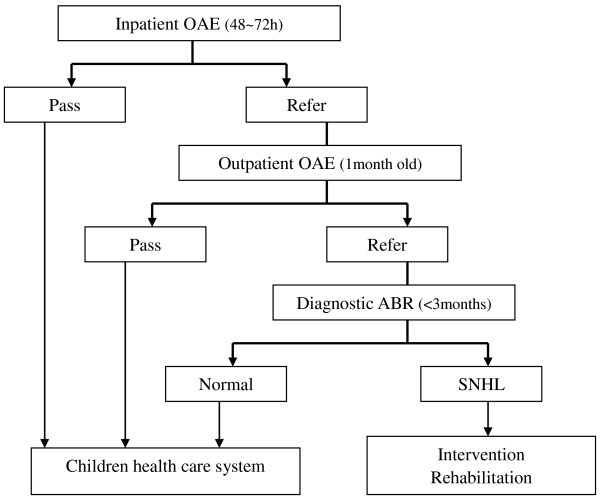
The currently recommended UNHS protocol.

Universal newborn hearing screening (UNHS) is an effective way of identifying hearing loss in newborns [10], and in conjunction with initiation of appropriate intervention within 6 months of diagnosis of hearing loss in infants shown to result in the development of significantly better language abilities, compared with infants identified with hearing loss later on in life [9,11]. Consequently, an increasing number of countries, including China, have incorporated the UNHS program into their public health systems. In China, a directive from the Beijing Municipal Government has been in place since July 2003, which requires all hospitals with an obstetrical department to implement a mandatory UNHS program (Figure 1) for all newborn babies in Beijing’s urban or rural areas [12,13]. Despite a political drive to intensify and expand the hospital-based newborn hearing screening, diagnosis, and intervention services [14], there has been disparity in the overall screening rates for hearing loss in newborn babies in urban (95.4%) or rural (84.1%) areas of Beijing, and likely other cities, over the last decade [15]. Whilst differences in economic development and health resources are likely to contribute to this difference in screening rates between the urban and rural areas, it is likely that migration from the rural to urban areas associated with the economic development also plays a major role. This is reflected by a 10-fold increase in the number of babies born to migrant mothers in 2007 (80,000 babies) compared with 1995 (8,000 babies) [16]. In view of this dramatic demographic change, the internal migrants (also known as “floating population”) have become increasingly relevant to the health care system, especially as they lose their government health care insurance when they leave their local areas. Moreover, frequent migration from one job to another has led to many pregnant migrants being examined in prenatal clinics in several hospitals. This in turn leaves them without consistent or structured pre-natal education or understanding of the importance of screening newborns for impaired hearing and the interventions available for impaired hearing.

Despite the relatively high socioeconomic impact of the internal migrant sub-population in China, to our knowledge no study has reported the screening outcomes including the drop-out rates among this sub-population, using the recommended UNHS protocol for China. We have presumed that although the currently recommended UNHS protocol is suitable for the urban resident population, it is not optimal for internal migrants for many of the reasons stated above. The aim of our study was thus to design a specific UNHS protocol based on the local social characteristics of internal migrants, such that it would improve the screening rates for hearing loss in their newborn babies. This protocol would additionally allow detection of infants suspected to have permanent childhood hearing impairment (PCHI), in order that appropriate intervention and rehabilitation can be provided at an early stage.

## Methods

### Participants

A total of 10983 babies (6048 males and 4935 females), born at Beijing’s Shangdi Hospital between January 2007 and December 2009, were investigated. All babies were from migrant families (parents neither have registered permanent residence nor stable work in Beijing) and appeared to be normal and healthy at birth.

### Hearing impairment testing protocol

**Figure 2:**
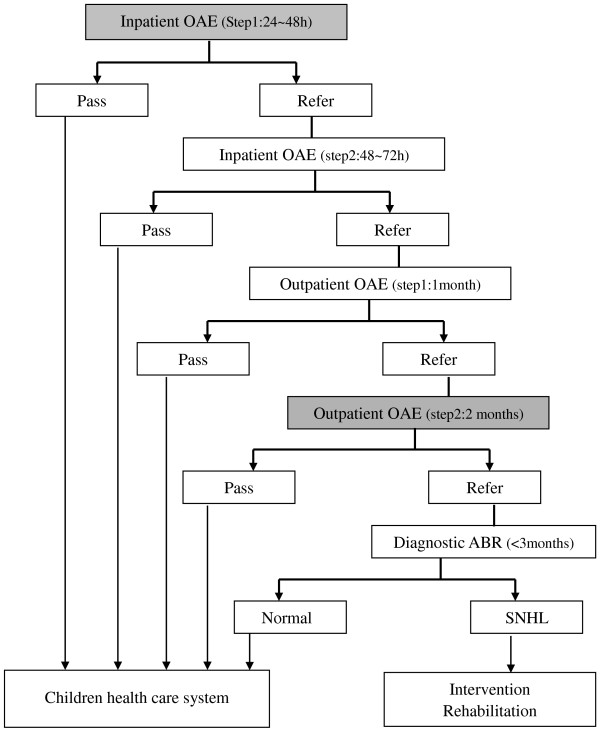
**Proposed revised UNHS protocol.** Steps added to currently recommended protocol are shaded grey.

Testing for hearing impairment was performed according to a modification of the recommended UNHS procedure, which incorporated an additional inpatient OAE test 24–48 hours after birth and an additional outpatient OAE test at 2 months after birth (Figure 2).

Briefly, all newborn babies underwent the inpatient otoacoustic emissions (OAE) test 24–48 hours after birth. When a baby tested positive and could stay in the hospital beyond 48 hours, it was referred to a second OAE test 48–72 hours after birth (this is the inpatient screening time in the recommend protocol). When a baby tested positive at the first test and did not undergo the second test, or when a baby tested positive at both tests as an inpatient, it was referred for an outpatient OAE test, at 1 month after birth. All infants testing positive after 1month were referred to an additional OAE test at 2 months after birth. Irrespective of whether or not an infant had undergone the OAE test after 2 months, all infants testing positive after 1 month were referred to a specialist hospital for an auditory brainstem response (ABR) test, for confirmation of a diagnosis of hearing impairment. When an infant was identified as having abnormal ABR, a further series of audiology tests; including OAE, ABR (AC and BC), 1 kHz tympanometry and CT (if necessary); were performed before 6 months of age, and appropriate intervention and rehabilitation were suggested to his/her parents/guardians.

### Methods

One of six qualified audiologists performed the first screening 24 to 48 hours after birth using the Transient Evoked Otoacoustic Emissions (TEOAE) test in the well-baby nursery. A Capella OAE equipment (Madsen, Denmark) presenting nonlinear clicks at 75dBpeak equivalent sound pressure level (SPL) was employed for this test. The TEOAE test was post-windowed by a 3 ms windowing function to suppress muscular and respiratory artefacts. Each recording was the mean result of 2080 sweeps; with minimal pass criteria being 50% reproducibility, 10dB SPL at emission response, and 3dB signal-to-noise ratio at any 3 analysis frequencies (1k, 1.5k, 2k, 3k, and 4 kHz). All OAE recordings were performed in a sound shielded room during the baby’s natural sleep, after the baby had been fed.

A diagnostic assessment for impaired hearing was finally performed by auditory brainstem response (ABR) testing, using an ICS Charter Evoked Potential equipment (GN, Denmark) in an electrical- and sound-shielded room. The settings employed for the ABR test were as shown in Table 1. Stimulus calibration for the test was performed by the “real-ear” method, based on mean detection threshold of 30 dB nHL (normal hearing level) clicks for 30 adults (18~25 years of age) with otologically normal hearing. The hearing loss was categorized by ABR wave V latency as mild (31-50dB nHL), moderate (51-70dB nHL), severe (71-90dB nHL), or profound ≥91dB nHL).

**Table 1:** The parameters and settings of ABR test

**Parameters**	**Settings**
Transducer	*Insert phone*
Polarity	*Alternating*
Stimulate	*Click (duration 100us)*
Sweep times	*15ms*
Rate	*21.1/sec*
Filter	*High pass 100Hz; Low pass 3kHz*
Gain	*100k*

All procedures were performed with the consent of the parent/guardian. The study protocol was approved by the Ethics Committee of Beijing Institute of Otolaryngology and performed in accordance with the guidelines of the World Medical Association’s Declaration of Helsinki.

### Statistical methods

Statistical analysis was performed using the SPSS software (V.13.0; SPSS Inc., USA). Chi-square test was used to detect significant differences in pass/refer rate between different steps of UNHS, and two-sided *p* values < 0.05 were considered to be statistically significant.

## Results

### Positive detection rate for inpatient hearing screening using OAE tests

**Figure 3:**
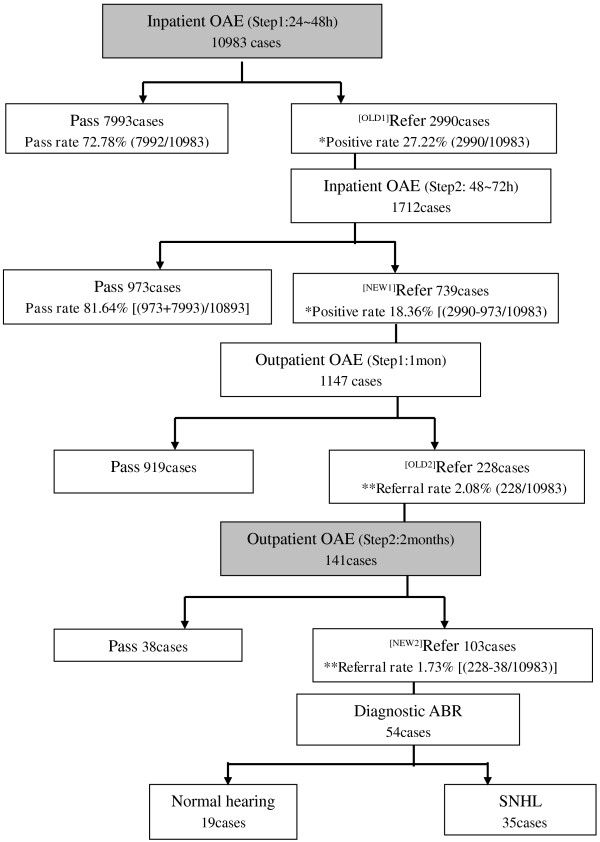
**Results of proposed revised UNHS protocol.** Steps added to currently recommended protocol are shaded grey. Additional steps, not found in the currently recommended protocol, are shaded gray. The comparable results were paired by label OLD and NEW in the flow chart.

A total of 98.91% (10983/11104) of newborn babies were tested for hearing impairment at the first inpatient screening, ≤48 hours after birth. Overall, 72.78% (7993/10983) of the babies passed newborn hearing screening, whereas 27.22% (2990/10983) of the babies were referred for a second inpatient hearing test 48–72 hours after birth. The parents/guardians of 57.25% (1712/2990) of these babies agreed to the second inpatient OAE test, following which 56.83% (973/1712) passed; thus significantly decreasing the overall positive rate from 27.22% (2990/10983) to 18.36% ([2990–973 /10983)]; χ^2^=244.906, p = 0)) (Figure 3).

### Referral rate for hearing screening test

Of the 2017(2990–973) infants suspected to have a hearing impairment 48–72 hours after birth, only 1147 (56.87%) underwent the OAE hearing test again after1-month at the hospital of their birth. Of these, 919 (80.12%) infants passed the test whereas 228 (19.88%) tested positive again and were referred for an additional OAE test at the same hospital at 2 months of age (i.e. overall referral rate of 2.08% (228/10983) at 1month of age). 141 of these 228 infants accepted the referral, of whom103 (73.05%) tested positive again by OAE test and ***were*** thus referred to a final confirmatory click ABR hearing impairment test (i.e. the final referral rate of 1.73% ([228-38/10983] at 2 months of age; χ^2^= 4.013, *p *= 0.045 < 0.05) vs. referral rate of 2.08% at 1month of age) (Figure 3).

Overall, of the 190 infants referred, parents and guardians of 84 agreed for their children to undergo the final ABR test at 2 months after birth. Only 54 of these infants attended the test and 35 were confirmed positive for impaired hearing. Of these 35 infants SNHL, 12 had bilateral hearing loss ranging from mild to profound and 23 had unilateral hearing loss (Table 2).

**Table 2:** The incidence of congenital hearing impairment in present study

**Degree of hearing loss**	**Bilateral HL (case)**		**Unilateral HL (case)**		**Total**
	**Symmetric**	**Asymmetric**	**Left ear**	**Right ear**	
Mild (31-50dB nHL)	6	2	8	7	23
Moderate(51-70dB nHL)	2	0	0	0	2
Severe(71-90dB nHL)	0	0	1	0	1
Profound(≥91dB nHL)	2	0	5	2	9
Total	10	2	14	9	35

Based on these findings, the prevalence of hearing loss in newborns of migrants in Beijing was found to be 0.32% (35/10983).

## Discussion

Many countries have adopted early hearing detection programs over the past 30 years; with several UNHS protocols such as single OAE and ABR screening, OAE followed by ABR, or AABR (Auto ABR) with a follow-up ABR, etc. being employed [17]. In China, most hospitals follow the OAE screening followed by a final diagnosis using the ABR test, as recommended by the US National Institute of Health (NIH) in 1993 [3]. This two-stage protocol is effective and has a low failure rate [18].

In the current investigation we modified this UNHS protocol to better address the needs of newborn babies of internal migrants, who have steadily increased in numbers over the last decade in Beijing and often miss out on this important screening service due to the geographic instability resulting from their parents’ way of life. In particular postpartum, families are often unable to accept regular hospital appointments due to their frequent moves, and are reluctant to commit themselves to rescreening tests at the same hospital where the baby was delivered, despite availability of free screening/rescreening tests. Indeed, a relatively lower rescreening rate of 56.87% in the current study was attributable to three major factors; namely i) some babies could not be rescreened because they had been sent back to their parent’s home provinces after a few days, ii) some babies were rescreened in hospitals closer to the their new rented accommodation and relevant data were not available to the investigators at Shangdi Hospital, and iii) some parents were reluctant to accept re-testing because their new rental location was far away. These findings thus emphasize the importance of revising currently recommended hearing screening protocols for newborn babies of frequently migrating parents. Based on previous research [19], our revised protocol calls for two inpatient OAE tests and two outpatient OAE tests instead of one each as recommended currently. These extra tests provide more scheduling flexibility and thus greater opportunity for newborns of migrant workers to be tested.

Using this modified protocol, we have demonstrated that there was increased coverage rate in the target population, the basic measure of screening efficiency. In a hospital such as Shangdi Hospital, where the majority of pregnant women are poor migrants without any form of health insurance and therefore eager to leave as soon as possible to avoid the expense of hospital stay, we shortened the inpatient screening time from the normal 72 hours to a maximum 48 hours by adding an earlier OAE screening test at 24-48 hours to ensure higher coverage/acceptance rates for hearing screening. Indeed, on reviewing our database we found that 2169 infants would have missed the inpatient hearing screening based on the recommended protocol. Moreover, as 492 of these infants did not pass the inpatient hearing screening at 24–48 hours, under the recommended protocol these infants with suspected hearing impairment would have been missed if they had left the hospital by about 48 hours. Using this modified protocol, the coverage rate was found to be high at 98.91%, meeting the recommended coverage by the Joint Committee of Infant Hearing (JCIH) [20], and was similar to the coverage rate observed for an obstetric hospital downtown (98.85%), where the majority of expectant mothers were residents [21]. The positive rate of 27.22% for hearing anomalies observed after the first hearing screening in the present study was much higher than that reported previously in several studies [21-24]. One study indicated that OAE testing had a 15.6% false positive rate in the first 24 hours of life, but this fell to 4% by 72 hours [22]. It is possible that the comparatively high rate for detection of hearing anomalies at the first screen in the current study was at least partly due to a high false positive rate, as most of the infants received their first OAE screening before they were 72 hours old; which has been suggested to be the ideal screening time [22].

In the modified UNHS protocol we kept the recommended inpatient OAE test at 48-72hours for referred infants staying in the hospital beyond 48 hours, to decrease the false positive rates. Although the present study indicated that the positive rate decreased significantly from 27.22% to 18.36% after the second inpatient OAE test 48–72 hours after birth, this was still much higher than the rate of 6.39% observed at the obstetric hospital downtown [21]. This disparity may be a result of not all infants being rescreened at the same time point of 72 hours, and suggests that selection of an appropriate screening/rescreening time following birth may be an important factor in minimising false positive rates for hearing anomalies in newborn babies.

The referral rate is another important measure of a screening program’s efficiency and effectiveness and, according to the JCIH 2007 position paper, can be minimised by effective rescreening [25]. Indeed, many well-infant screening protocols incorporate an outpatient rescreening within 1 month of hospital discharge to minimize the number of infants referred for follow-up audiological and medical evaluation. Some studies have demonstrated that following auditory neurological maturity and transient middle ear effusion absorption, some infants referred for outpatient OAE screening at 1 month passed the OAE test at 2 months [26,27]. In this context we felt that a single outpatient rescreening test after 1-month of birth was not appropriate for a migrating population for reasons discussed above, and therefore modified the UNHS protocol to include a second outpatient rescreening test at 2 months to increase the possibility for a greater number of migrants being able to accept at least one OAE test before further audiological assessment. Using the modified protocol, we found that the final referral rate for ABR testing was significantly reduced from 2.08% to 1.73%.

Of the 10983 migrant newborn babies that had the first hearing screening test, only 0.32% (35/10983) was found to have SNHL. The proportion of infants with identified unilateral (n = 23) to bilateral (n = 12) hearing loss in our study was similar to that previously reported in developing countries; approximately 1:3–4 [23,28,29]. However, if we include the missing subjects, we estimate that the total incidence of congenital SNHL would be 1.06%. It is important to note, that our study missed all infants with neural hearing loss (auditory neuropathy) because the current screening program relies on OAE, which assesses pre-neural functioning. The prevalence of sensory and neural hearing losses would therefore exceed the current estimated. Further, over time, late-onset and progressive hearing losses will increase the number of children who would benefit from intervention [30].

## Conclusion

Although rescreening tests are the most important method for decreasing the false positive and referral rates, low rescreening rates have generally been a problem in newborn hearing screening programs [24,28,29], and even more so for frequent migrants. Our study shows that it is feasible and practical to achieve high coverage rates for screening for hearing loss and decrease the referral rates in newborn babies of migrant workers, using a modification of the currently employed UNHS protocol. It is likely that further modifications and better design of the UNHS protocol reflecting advantageous socioeconomic factors specific to particular populations in different parts of the world, as well as employment of stringent patient follow-up systems and data management may significantly improve the screening/rescreening and referral rates for hearing loss in newborn babies.

## Competing interests

The authors report no conflicts of interest.

Further modifications and better design of the UNHS protocol reflecting socioeconomic factors specifically advantageous to particular populations may lead to significantly improved referral and rescreening.

## Authors’ contributions

BQ collected and analyzed the data and contributed to writing the paper. XC collected, analyzed the data. HE collected the data. BL designed the study. SP collected the data. YZ collected the data. ZC collected the data. LH designed the study and analyzed the data. LZ designed the study, analyzed the data and contributed to writing the paper. DH designed the study. All authors read and approved the final manuscript.

## Pre-publication history

The pre-publication history for this paper can be accessed here:

http://www.biomedcentral.com/1471-2431/13/116/prepub
